# CT Imaging Assessment of Pancreatic Adenocarcinoma Resectability after Neoadjuvant Therapy: Current Status and Perspective on the Use of Radiomics

**DOI:** 10.3390/jcm12216821

**Published:** 2023-10-29

**Authors:** Hala Khasawneh, Hanna Rafaela Ferreira Dalla Pria, Joao Miranda, Rachel Nevin, Shalini Chhabra, Dina Hamdan, Jayasree Chakraborty, Tiago Biachi de Castria, Natally Horvat

**Affiliations:** 1 Department of Radiology, University of Texas Southwestern, 5323 Harry Hines Blvd, Dallas, TX 75390, USA; hala.khasawneh@utsouthwestern.edu; 2 Department of Radiology, University of Iowa, 200 Hawkins Dr, Iowa City, IA 52242, USA; hanna-dallapria@uiowa.edu; 3 Department of Radiology, Memorial Sloan Kettering Cancer Center, 1275 York Avenue, New York, NY 10065, USA; santosj2@mskcc.org (J.M.); rachel.nevin90@gmail.com (R.N.); chhabras@mskcc.org (S.C.); 4 Department of Radiology, University of Sao Paulo, R. Dr. Ovidio Pires de Campos, 75-Cerqueira Cesar, Sao Paulo 05403-010, SP, Brazil; 5 Department of Radiology, The Mount Sinai Hospital, 1468 Madison Ave, New York, NY 10029, USA; dina.hamdan@mountsinai.org; 6 Department of Surgery, Memorial Sloan Kettering Cancer Center, 1275 York Avenue, New York, NY 10065, USA; chakrabj@mskcc.org; 7 Department of Gastrointestinal Oncology, Moffit Cancer Center, 12902 USF Magnolia Drive, Tampa, FL 33612, USA; tiago.biachi@moffitt.org; 8 Morsani College of Medicine, University of South Florida, 4202 E. Fowler Avenue, Tampa, FL 33620, USA

**Keywords:** pancreatic cancer, neoadjuvant therapy, computed tomography, treatment response, radiomics

## Abstract

Pancreatic adenocarcinoma (PDAC) is the most common pancreatic cancer and is associated with poor prognosis, a high mortality rate, and a substantial number of healthy life years lost. Surgical resection is the primary treatment option for patients with resectable disease; however, only 10–20% of all patients with PDAC are eligible for resection at the time of diagnosis. In this context, neoadjuvant therapy has the potential to increase the number of patients who are eligible for resection, thereby improving the overall survival rate. For patients who undergo neoadjuvant therapy, computed tomography (CT) remains the primary imaging tool for assessing treatment response. Nevertheless, the interpretation of imaging findings in this context remains challenging, given the similarity between viable tumor and treatment-related changes following neoadjuvant therapy. In this review, following an overview of the various treatment options for PDAC according to its resectability status, we will describe the key challenges regarding CT-based evaluation of PDAC treatment response following neoadjuvant therapy, as well as summarize the literature on CT-based evaluation of PDAC treatment response, including the use of radiomics. Finally, we will outline key recommendations for the management of PDAC after neoadjuvant therapy, taking into consideration CT-based findings.

## 1. Introduction

Pancreatic cancer is the tenth most common cancer in the United States, with an estimated 64,050 new cases in 2023. While it is only the tenth most common cancer, it is the third leading cause of all cancer-related deaths in the United States, being responsible for an estimated 50,550 deaths in 2023 [1,2,3]. Moreover, the number of deaths attributed to pancreatic cancer is projected to rise even more through the next 20 years, and predicted to be the second leading cause of all cancer-related deaths in the United States by 2040 [1]. Similarly, according to GLOBOCAN 2020, pancreatic cancer is the fourteenth most common cancer globally but is the seventh leading cause of all cancer-related deaths [4], and according to a study across 28 European Union countries, it is projected to be the third leading cause of all cancer-related deaths in this region by 2025 [5]. In addition, it is also responsible for a substantial number of healthy life years lost; for example, approximately 9.1 million quality-adjusted life years were lost due to pancreatic cancer globally in 2017 [6].

Of all pancreatic cancer types, pancreatic ductal adenocarcinoma (PDAC) type accounts for the majority of cases (85–90%) [7]. PDAC is associated with poor prognosis and, correspondingly, a high mortality rate [8]. Therefore, PDAC in particular is a significant global burden of disease. While surgical resection is the primary treatment option for patients with resectable PDAC, only 10–20% of all patients with PDAC are eligible for resection at the time of diagnosis [9]. Notably, neoadjuvant therapy has the potential to increase the number of patients who are eligible for resection, thereby improving the overall survival rate [10]. However, in patients with resectable and borderline resectable PDAC in particular, the indications of neoadjuvant therapy remain debated in the literature, with neoadjuvant therapy currently being used in these patients depending on their clinical characteristics and the experience of the multidisciplinary disease management team.

For the staging, restaging, and follow-up of patients with PDAC, diagnostic imaging remains the primary tool, with contrast-enhanced computed tomography (CT) being the most commonly used imaging modality for the local assessment of PDAC. In the primary staging setting, imaging is indicated to classify the tumor as resectable, borderline resectable, locally advanced, or metastatic [11,12]. In the restaging setting, e.g., after neoadjuvant therapy, imaging is indicated for response assessment, usually CT. Nonetheless, the interpretation of imaging findings in this setting remains challenging; for CT in particular, it remains difficult to distinguish between viable tumor and treatment-related alterations such as inflammation, edema, and fibrosis following neoadjuvant therapy [13].

In this review, following an overview of the various treatment options for PDAC according to its resectability status, we will dive into the imaging-based assessment of PDAC following neoadjuvant therapy. Specifically, as CT remains the primary imaging modality for the evaluation of PDAC, we will describe the key challenges regarding CT-based evaluation of PDAC treatment response following neoadjuvant therapy, as well as summarize the literature on CT-based evaluation of PDAC treatment response, including the use of radiomics. Finally, we will outline key recommendations for the management of PDAC after neoadjuvant therapy, taking into consideration CT-based findings.

## 2. Overview of the Treatment of PDAC

**Figure 1:**
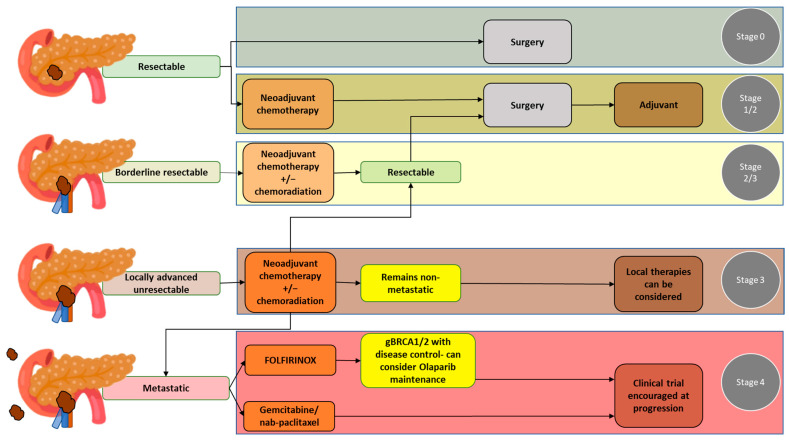
Pancreatic ductal adenocarcinoma groups according to resectability status and the most common treatment options for each group.

Treatment plans for PDAC should be made following a comprehensive evaluation, including primary staging of the disease and assessment of the patient’s overall health status. At primary staging, PDAC is classified as a resectable disease, borderline resectable disease without metastases, locally advanced disease, or metastatic disease. Accuracy in the primary staging setting is crucial, as disease staging significantly affects treatment decisions. Guidelines for the management of pancreatic cancer have been published by the National Comprehensive Cancer Network (NCCN) in the United States [14]. Of note, considering the complexity of PDAC, the NCCN also strongly recommends that the management of patients with PDAC should involve high-quality imaging and multidisciplinary discussion at a high-volume center [14]. The NCCN guidelines pertaining to the resectability of pancreatic cancer, which are based on vascular involvement, are widely accepted and used at multidisciplinary team discussions in the United States [14,15] (Table 1). Below, the different PDAC resectability groups and the most common treatment options for each group are detailed (see also Figure 1 for a summary).

### 2.1. Resectable PDAC

**Figure 2:**
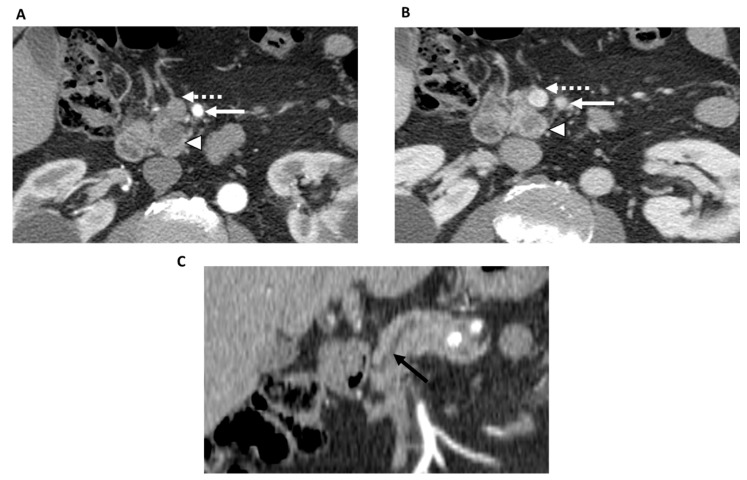
78-year-old man with resectable pancreatic adenocarcinoma within the uncinate process. Contrast-enhanced CT in the axial plane shows a tumor within the uncinate process (arrowhead) in both the arterial (**A**) and portal venous phases (**B**). The superior mesenteric artery (white solid arrow) and superior mesenteric vein (dashed arrow) are not involved by the tumor. Contrast-enhanced CT in the coronal plane (**C**) shows upstream main pancreatic duct dilatation (black arrow). There was no evidence of metastatic disease. Based on the NCCN criteria, the patient was classified as resectable and underwent upfront surgery with R0 resection.

Surgery is the primary treatment option for patients with resectable PDAC. The goal of surgery is to obtain R0 resection, which entails the complete resection of the primary tumor and regional lymph nodes, since positive margins are associated with poor long-term survival [16,17] (Figure 2). After surgery, adjuvant chemotherapy is indicated. Of note, while surgical resection and adjuvant chemotherapy can be beneficial for improving survival outcomes, the median survival ranges from 20 to 53.5 months even under optimal conditions [14,18,19]. Some studies have shown that neoadjuvant therapy followed by surgery and adjuvant therapy further improves overall survival [20]; thus, neoadjuvant chemotherapy may also be considered in these patients.

### 2.2. Borderline Resectable PDAC

Considering the complexity of borderline resectable PDAC, multidisciplinary discussion between medical oncologists, radiologists, surgeons, and radiation oncologists is especially necessary to determine the best treatment strategy. Treatment options include upfront surgery as well as neoadjuvant chemotherapy with or without radiation therapy. It is also advisable to refer borderline PDAC patients to participate in a clinical trial, as clinical trials offer the opportunity to undergo innovative treatment approaches, new drug combinations, and emerging therapies that can potentially improve patient outcomes.

The goal of neoadjuvant therapy is the downstaging of the tumor, which improves the likelihood of an R0 surgical resection, thereby prolonging survival rates; in this way, neoadjuvant therapy can also identify patients with rapid progression and poor response to treatment [21,22]. While the comparison between neoadjuvant and adjuvant therapies is challenging due to the different patient populations that receive these therapies, recent systematic reviews and meta-analyses underscore the benefits of neoadjuvant therapy over adjuvant therapy, including improved overall survival, higher R0 resection rates, and fewer pathological lymph nodes [23,24,25]. Further, with the literature suggesting an increasing shift towards adopting a neoadjuvant approach, numerous ongoing trials are examining the effectiveness of different neoadjuvant chemotherapy approaches, such as neoadjuvant therapy involving FOLFIRINOX, neoadjuvant therapy involving gemcitabine/nab-paclitaxel, and neoadjuvant therapy combining chemotherapy and radiation therapy [26]. Promising outcomes from neoadjuvant therapy involving chemotherapy indicate that chemotherapy should be an integral component of the neoadjuvant strategy. The use of stereotactic radiotherapy and emerging techniques like proton therapy are also being explored as part of the neoadjuvant strategy, with encouraging results [27].

Given the various neoadjuvant strategies, molecular profiling at diagnosis and the evaluation of the immune environment are emerging as potential tools to personalize neoadjuvant strategies. Encouragingly, combining neoadjuvant therapy with immunotherapy has shown promise to improve patient outcomes, particularly in the context of localized disease [26].

### 2.3. Locally Advanced PDAC

**Figure 3:**
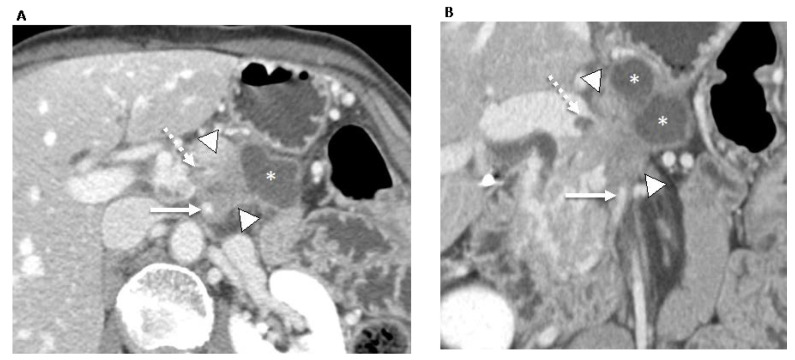
70-year-old man with locally advanced pancreatic ductal adenocarcinoma. Contrast-enhanced CT in the axial (**A**) and coronal (**B**) planes in the portal venous phase shows an ill-defined pancreatic neck and body tumor (arrowheads) with an adjacent pseudocyst (asterisks). The tumor involves the locoregional vessels including encasement (>180°) of the superior mesenteric artery (white arrow) and superior mesenteric vein/portomesenteric junction, which was severely narrowed with small non-occlusive thrombus (dashed arrow). The patient underwent palliative radiotherapy.

For patients with locally advanced PDAC and poor performance status, treatment options include best supportive care or palliative care (e.g., palliative radiotherapy, chemotherapy using a single agent, or polychemotherapy) (Figure 3). For patients with locally advanced PDAC and good or intermediate performance status, treatment options include systemic chemotherapy, chemoradiation therapy, or therapy as part of a clinical trial. Among the patients with locally advanced PDAC and good or intermediate performance status, if there is no disease progression, surgical resection may also be feasible [14].

### 2.4. Metastatic PDAC

For patients with metastatic PDAC, treatment options include systemic chemotherapy (e.g., involving FOLFIRINOX or gemcitabine with nab-paclitaxel), targeted therapy, or palliative care. Treatment for these patients is aimed towards managing the disease and improving their quality of life. The choice of treatment will depend on the patient’s performance status, the extent of the disease, and the patient’s treatment response.

## 3. Challenges to CT-Based Assessment of PDAC Treatment Response following Neoadjuvant Therapy

For the evaluation of treatment response following neoadjuvant therapy in patients with PDAC, CT remains the most commonly used imaging modality as it offers several advantages over the other imaging modalities, including higher spatial resolution and multiplanar reconstruction capability [9]. However, CT has low accuracy in predicting R0 resection after neoadjuvant therapy, since CT cannot accurately distinguish between residual tumor and tissue scarring after tumor regression [16]. Further, local inflammatory pancreatitis cannot be distinguished from tumor infiltration, which can lead to the under-estimation of tumor resectability [17,28,29]. These limitations are compounded by the fact that only a few patients show tumor shrinkage after neoadjuvant therapy, and most patients have stable disease [30]. Considering all these points, most borderline patients are eligible for R0 resection even though they do not show a radiological response [31]. This highlights the challenges in assessing the efficacy of current treatments and the importance of exploring alternative strategies to improve patient assessment and outcomes.

## 4. Literature Search Strategy

To identify pertinent articles and studies, a comprehensive search strategy was employed. Utilizing the PubMed database, the following search terms were used: “Pancreatic Adenocarcinoma”, “CT Imaging”, “Neoadjuvant Therapy”, “Radiomics”, and “Resectability”. Boolean operators (AND, OR) and quotation marks were strategically applied to optimize the search results. The search was performed without a specified date limit to encompass the breadth of the available literature. Subsequently, the results were reviewed, and relevant articles were selected for inclusion in the study, providing a foundation for the literature review and background of this research endeavor.

## 5. Semantic CT Imaging Features for the Assessment of PDAC Treatment Response after Neoadjuvant Therapy

Only a few studies have assessed the use of semantic CT imaging features (i.e., imaging features that can be seen visually by the radiologist) to evaluate treatment response following neoadjuvant therapy in patients with PDAC (Table 2). To date, these studies have originated from several countries, including the United States, South Korea, France, Portugal, and Italy. The largest study had a sample size of 343 patients, while the smallest had a sample size of 36 patients. Patients received different types of neoadjuvant therapy, with some undergoing chemotherapy alone and some undergoing chemoradiation therapy; however, so far, no study has investigated the differences in CT imaging findings between these two types of neoadjuvant therapy.

Semantic CT imaging findings that have been investigated include tumor size, volume, diameter, or attenuation, including those assessed according to RECIST criteria [31,33,34,36,38,39]; the product of three axes [34]; radiological tumor stage according to American Joint Committee on Cancer (AJCC) criteria [38]; vascular contact/involvement [33,34,40]; tumor response according to RECIST criteria [35,36,39]; resectability status including resectability according to NCCN or Americas Hepato-Pancreato-Biliary Association (AHPBA) guidelines [13,36,37]; NCCN resectability criteria, e.g., contrast enhancement of the soft tissue and veins [40]; tumor–pancreas interface response based on a classification developed by a group of investigators [35]; and resectability status based on institutional criteria [32].

**Figure 4:**
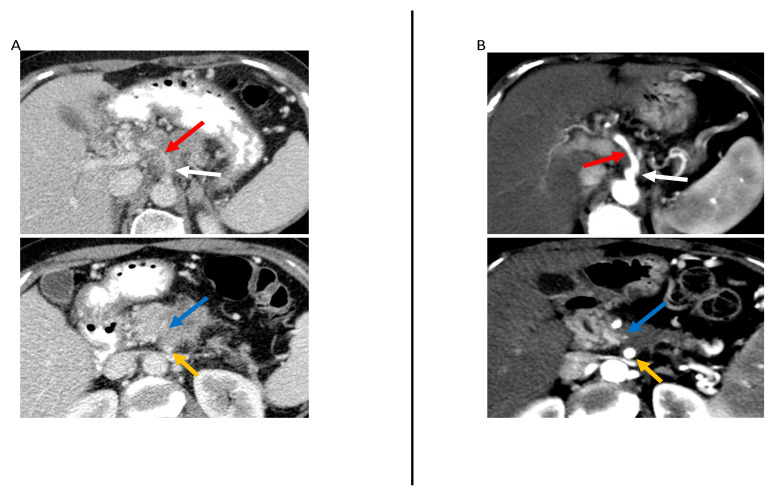
59-year-old woman with locally advanced pancreatic cancer before and after neoadjuvant therapy. Contrast-enhanced CT in the axial plane at primary staging (**A**) and restaging after neoadjuvant therapy (**B**) demonstrate an ill-defined pancreatic neck and body tumor (with markedly decreased size after neoadjuvant therapy (blue arrow). At primary staging, the tumor was abutting the celiac artery (white arrow) superior mesenteric artery (SMA) (yellow arrow), causing vessel irregularity and narrowing (red arrow). After neoadjuvant therapy, the SMA abutment by the tumor was overall unchanged, although the artery irregularity and narrowing resolved. Additionally, the soft tissue surrounding the SMA had an enhancement < 30 HU. The patient underwent surgical resection after neoadjuvant therapy and there was no evidence of arterial involvement.

Overall, changes in tumor size or volume following neoadjuvant therapy were not significantly associated with R0 resection or survival outcomes. The exception was Perri et al. [39], who found that a reduction in tumor volume as well as partial response (as defined by RECIST 1.1 guidelines) were significantly associated with major pathologic response, defined as <5% viable cancer cells in the surgical specimen (*p* < 0.01 for both) (Figure 4).

Regarding tumor attenuation, Marchegiani et al. [36] found that increased tumor attenuation in the arterial and venous phases after neoadjuvant therapy was associated with R0 resection in patients with locally advanced and borderline resectable tumors (*p* < 0.001 and *p* = 0.001, respectively). However, in Cassinotto et al. [33] and Wagner et al. [34], attenuation was not associated with R0 resection.

Regarding vascular contact/involvement, Cassinotto et al. [33] demonstrated that partial regression of tumor–vessel contact after neoadjuvant therapy had 100% positive predictive value for R0 resection, regardless of the degree of either reduction in tumor size or residual vascular involvement (Figure 4). Otherwise, studies showed that vascular contact/involvement was not associated with R0 resection [34,40].

Low contrast enhancement of the soft tissue contacting the artery (≤46.4 HU) was shown by Jang et al. [40] to be associated with R0 resection (adjusted odds ratio = 7.4; *p* = 0.01). Jang et al. also found that regression or stability of the NCCN resectability status after neoadjuvant therapy was associated with improved recurrence-free survival (Figure 4).

Amer et al. [35] classified the response of the tumor–pancreas interface as a type I response (interface remaining unchanged or becoming more defined) and type 2 response (interface becoming less defined). A type 1 response was associated with pathological complete response or near complete response (*p* = 0.01), leading to increased disease-free survival and overall survival.

Finally, Kim et al. [32] used their institutional criteria to determine resectability, whereby resectable tumors were defined as having no distant metastases, no paraaortic nodal metastasis (>1 cm in short axis), and no evidence of invasion of the celiac axis, superior mesenteric artery, hepatic artery, or superior mesenteric vein–portal vein confluence. Arterial invasion was defined as any direct tumor-to-vessel contiguity, even if it was <50%, and venous invasion was defined as tumor-to-vessel circumferential contiguity > 50% and invasion > 2–3 cm in length. Resectability status according to these institutional criteria demonstrated an accuracy of 83%.

## 6. Radiomics as a Valuable Tool to Aid in the Assessment of PDAC Treatment Response after Neoadjuvant Therapy

As can be seen above, the use of semantic CT imaging features in the assessment of treatment response and resectability in PDAC after neoadjuvant therapy remains limited. Meanwhile, radiomics has emerged as a valuable tool that involves extracting and analyzing quantitative imaging features from medical images, offering the potential to enhance treatment response assessment [41]. In the context of treatment response assessment, while radiologists may qualitatively describe PDAC enhancement patterns, vascular involvement, and the tumor–parenchyma interface, radiomics can capture subtle quantitative differences not seen by the naked eye.

### 6.1. Pipeline of Studies Using Radiomics

**Figure 5:**
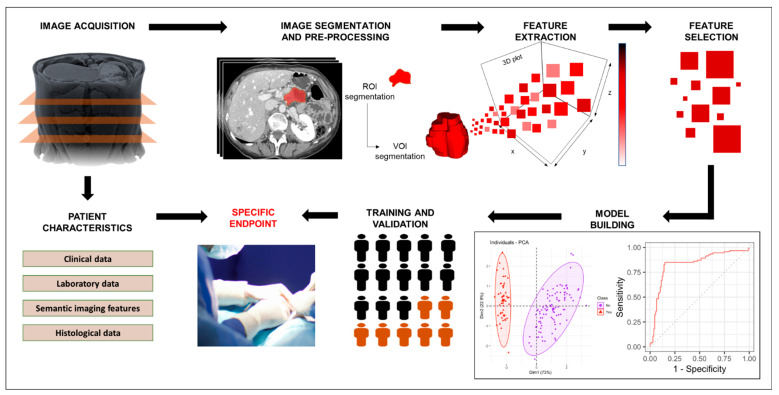
Radiomics pipeline. Abbreviations: *ROI:* region of interest; *VOI:* volume of interest.

Radiomics enables the quantitative assessment of subtle spatial variations pertaining to pixel intensities and distribution within radiological images; in this way, radiomics has the potential to provide insights into pathologic changes in the tumor and surrounding regions beyond what can be perceived by the naked eye alone [42,43]. Given this potential, many studies have been conducted in recent years to explore the use of radiomics. Such studies involve a multistep pipeline [44,45], as described below (also, see Figure 5). Of note, the first two steps are applicable to all research studies involving radiological imaging, i.e., not only those involving radiomics.

(a) *Pre-execution*: In this step, the research team determines the study design, the involvement of human subjects, the imaging modalities that will be used, and the clinically relevant endpoints that will be studied. This step aims to determine the feasibility of the study, including ensuring that sufficient high-quality data will be obtained.
(b) *Data curation*: In this step, clinical data, imaging data, and other relevant metadata are collected and managed according to the needs of the study. Of note, image anonymization is a key aspect of data curation; proper image anonymization is not only crucial to maintaining patient/participant privacy, but it is also an essential characteristic of high-quality data.
(c) *Image segmentation*: In this step, tumors, peritumoral or tumor subregions (also known as tumor habitats), 2D regions of interest, or 3D volumes of interest are segmented, whether manually, automatically, or semi-automatically. This step aims to provide a representative area or volume to be assessed.
(d) *Image pre-processing*: This step involves the application of a filter, normalization, resampling, and/or thresholding, aiming to increase standardization and/or improve image quality.
(e) *Feature extraction*: In this step, different classes of features are extracted from the segmented area or volume, including morphological features (i.e., visual characteristics and shape), first-order or histogram-based features (i.e., features describing the distribution of the pixel intensities within the segmented area), and second-order textural features (i.e., features capturing the spatial relationship between pixels intensities within the segmented area or volume).
(f) *Feature selection*: In this step, from all the extracted features, those features that are stable, informative, and non-redundant are selected for model building.
(g) *Model building*: Model building is a crucial step, in which a radiomic model is developed using the selected radiomic features to enable realistic and accurate diagnosis, prognosis, or response prediction. During this step, the comparison of the performance of the radiomic model against that of clinically used tools is helpful for determining the added value of the radiomic model.
(h) *Validation*: This step involves assessing the performance and generalizability of the developed radiomic model by applying it to an independent dataset, ideally from a different institution. This step ensures model robustness and effectiveness before potential real-world implementation.

### 6.2. Studies Applying Radiomics in the Assessment of PDAC Treatment Response after Neoadjuvant Therapy

While radiomics is a promising tool, to date, only a few studies have assessed the utility of CT-based radiomics (or CT texture analysis which entails some characteristics of radiomics), for the assessment of PDAC treatment response after neoadjuvant therapy (Table 3). These studies are predominantly from the United States, with additional contributions from South Korea and Italy. Moreover, it is relevant to note that none of these studies were multicenter in nature or involved external validation. The largest study had a sample size of 194 patients, while the smallest had a sample size of 20 patients.

Most studies involved manual segmentation of the pancreatic tumor. In Chen et al., radiomic features that were associated with a good pathologic response included changes in the mean histograms of CT number, standard deviation, skewness, and kurtosis [29]. Nasief et al. [47] combined delta radiomic features and the widely used clinical tool of CA 19-9 levels, which yielded a c-index of 0.87, which was significantly improved from that of CA 19-9 alone (c-index—0.69), highlighting the added value of radiomic features. Meanwhile, Ciaravino et al. investigated several texture features but found that only kurtosis was significantly different between primary staging and retagging CT (*p* = 0.0046) and that kurtosis was indicative of tumor downstaging [46]. Like Ciaravino et al., Borhani et al. [48] investigated several texture features, finding that higher mean perfusion parameter values at primary staging produced higher odds of a favorable pathologic response (OR = 1.06; 95% CI, 1.002–1.12); additionally, the Cox model containing three texture features was significantly associated with disease-free survival (*p* = 0.001). Kim et al. [37] also investigated several texture features; while they did not find any texture feature at primary staging that was associated with R0 resection, a few subtracted texture feature values (where values represent the difference between primary staging and restaging) were associated with R0 resection and/or overall survival.

One study by Rigiroli et al. [49] involved semi-automatic segmentation of 3D VOIs containing the tumor and perivascular tissue surrounding the superior mesenteric artery (SMA). The radiomic-based model containing five radiomic features extracted from the tumor and perivascular tissue had an AUC of 0.71 to determine SMA tumor involvement. This AUC was notably superior to the AUC of 0.54 achieved by NCCN resectability status determined through multidisciplinary review [49].

The precise pathologic correlates of the radiomic features that constitute the machine learning classifiers are not entirely known. The major limitations of these studies include their retrospective nature, rendering them prone to selection bias. Additionally, the number of subjects included in these studies are relatively small. Prospective studies with larger cohorts are warranted for further validation.

## 7. Current Recommendations for the Management of PDAC after Neoadjuvant Therapy

NCCN guidelines recommend that after neoadjuvant therapy, if there is no evidence of metastasis, the determination on whether to perform surgical resection after neoadjuvant therapy in patients with PDAC should be made through multidisciplinary discussion, taking into account imaging findings, cancer antigen (CA 19-9) levels, and clinical symptoms [14]. Regarding imaging findings, while soft tissue may increase after neoadjuvant therapy, it is not necessarily correlated with disease progression, and imaging findings tend to be stable between primary staging and restaging after neoadjuvant therapy [34].

For tumors classified as resectable or borderline resectable tumors at primary staging, exploratory surgery should be considered if there is no definite progression on imaging, and CA 19-9 levels either decreased or remained unchanged. Surgery may also be indicated if the portal vein or superior mesenteric vein are involved following neoadjuvant therapy, but are in the presence of patent vessels for vascular reconstruction proximally and distally to the site of the vessel involvement. Even in the presence of slightly increased perivascular soft tissue thickening, surgery may still be considered if this finding is accompanied by other signs of clinical improvement, such as an increased performance status and reduced symptoms (e.g., reduced abdominal pain).

Locally advanced tumors at primary staging can be considered for exploratory surgical resection following neoadjuvant therapy if there is significant clinical improvement, markedly decreased CA 19-9, and no definite tumor progression on imaging. Moreover, it is essential to have a comprehensive discussion with the patient regarding the advantages and disadvantages of the surgery. If the decision to proceed is made, it should be performed at highly specialized centers.

In addition to the NCCN guidelines, the American Journal of Roentgenology Expert Panel Narrative Review [50] presented several consensus points regarding the imaging assessment of PDAC after neoadjuvant therapy. Several of these points are summarized below:

- CT is currently not sufficiently accurate to predict R0 resection.
- Imaging frequently underestimates resectability following neoadjuvant therapy.
- Favorable imaging findings after neoadjuvant therapy include partial regression of tumor contact with peripancreatic vessels, a mild fat-stranding perivascular halo in place of solid tumor contact with a vessel, and reduction in tumor size according to RECIST 1.1 guidelines.
- Surgery after neoadjuvant therapy should be considered even if imaging findings are unchanged from those at primary staging.
- CT is more accurate for evaluating venous involvement than for arterial involvement after neoadjuvant therapy. Decreased venous stenosis or decreased contour deformation indicates improved venous involvement.

## 8. Conclusions

In conclusion, this review presents the current state of CT-based assessment of PDAC treatment response after neoadjuvant therapy and emphasizes the potential of radiomics as a complementary tool for CT-based assessment. While the current literature is lacking in studies investigating the added value of radiomics to improve the accuracy and reliability of CT-based assessment after neoadjuvant therapy, it is expected that more studies will be conducted in the future, including multicenter, prospective studies which are urgently needed to pave the way for the integration of radiomics into clinical practice, ultimately benefitting many patients with PDAC.

## Figures and Tables

**Table 1:** NCCN criteria regarding the resectability status of pancreatic adenocarcinoma at diagnosis (primary staging).

Resectability Status	Arterial	Venous
Resectable	No tumor contact	- No tumor contact with the SMV or PV - ≤180° tumor abutment without vein contour irregularity
Borderline Resectable	CA & branches: - Solid tumor contact with CHA without extension to the CA or hepatic artery bifurcation - Solid tumor abutment with CA of <180° SMA: - Solid tumor abutment with SMA of <180°	SMV or PV: - Solid tumor encasement with the SMV or PV of >180° - Solid tumor abutment of ≤180° with contour irregularity of the vein - Thrombosis of the vein but with suitable vessels proximal and distal to the site of involvement allowing for safe, complete resection and vein reconstruction. IVC: - Solid tumor contact with the IVC
Locally Advanced	- Solid tumor encasement >180° with the CA or SMA - Aortic involvement	- Unreconstructible SMV or PV due to tumor or bland thrombus

Abbreviations: *CA:* celiac axis; *CHA:* common hepatic artery; *IVC:* inferior vena cava; *PV:* portal vein; *SMA:* superior mesenteric artery; *SMV:* superior mesenteric vein.

**Table 2:** Summary of studies to date that have assessed the use of semantic CT imaging features to evaluate PDAC treatment response assessment following neoadjuvant therapy.

Author, Year (Country)	n	Imaging Criteria	Type of Neoadjuvant Therapy	No. and Type of Readers	Main Results
Tamm et al. [13], 2006 (U.S.)	55	-Resectability based on NCCN criteria	-CRT	3 radiologists	-Resectability as determined qualitatively by three radiologists had an accuracy of 87–95%.
Kim et al. [32], 2009 (South Korea)	38	-Institutional CT criteria **	-CRT	2 radiologists	-Presurgical CT interpreted based on institutional CT criteria had an accuracy of 83% (10/12), sensitivity of 91% (10/11), specificity of 0% (0/1), PPV of 91% (10/11), and NPV of 0% (0/1) for predicting resectability.
Katz et al. [31], 2012 (U.S.)	129	-Changes in tumor size or stage using RECIST 1.1 criteria	-Chemo -CRT -EBRT	1 GI radiologists	-Response according to RECIST was not associated with OS (*p* = 0.78)
Cassinotto et al. [33], 2014 (France)	47	-Tumor diameter -Tumor attenuation -Tumor vascular contact	-CRT	2 GI radiologists	-Partial regression of tumor contact with the SMV/portal vein had a PPV of 100% (10/10) for R0 resection. Partial regression of tumor contact with any peripancreatic vascular axis had a PPV of 91% (20/22) for R0 resection.
Wagner et al. [34], 2017 (France and Portugal)	36	-Morphologic criteria: Tumor size, large axial axis, small axial axis. height and product of the three axes, attenuation, and response according to RECIST criteria -Vascular involvement -NCCN classification	-Chemo -CRT	2 radiologists	-Only the large axis and the product of the three axes were significantly associated with R0 resection.
Amer et al. [35], 2018 (U.S.)	326	-Response according to RECIST 1.1 criteria -Tumor/pancreas interface response developed by the authors	-Chemo -CRT	3 radiologists	-Type I vs. Type II response at the interface was significantly associated with fewer viable cells after neoadjuvant therapy and was more likely to achieve major pathologic response (*p* = 0.01); Type I response also showed improved DFS and OS.
Marchegiani et al. [36], 2018 (Italy)	59	-Tumor attenuation -Longest tumor dimension -Response according to RECIST criteria -Resectability status according to the Americas Hepato-Pancreato-Biliary Association (AHPBA) guidelines	-Chemo -CRT	2 radiologists	-Only an increase in mean tumor attenuation in the arterial and venous phases following neoadjuvant therapy was significantly associated with R0 resection (*p* < 0.001 and 0.001 for the arterial and venous phases, respectively).
Kim et al. [37], 2019 (South Korea)	45	-Resectability (not indicated if specific criteria were used)	-Chemo -CRT	2 radiologists	-CT had 51–69% to predict R0 resection.
Wei et al. [38], 2021 (U.S.)	343	-Longest tumor diameter -Radiological tumor stage according to American Joint Committee on Cancer (AJCC) criteria -Tumor volume	-Chemo -CRT	2 radiologists	-Longest tumor diameter tends to understage ypT -Radiological tumor stage and tumor volume post neoadjuvant therapy were correlated with ypT stage, tumor response grades, distance of superior mesenteric artery margin, and tumor recurrence/metastasis.
Perri et al. [39], 2021 (U.S.)	290	-Response according to RECIST 1.1 -Other changes in tumor size and anatomic extent	-Chemo -CRT	1 surgeon	-RECIST partial response and reduction in tumor volume were significantly associated with major pathologic response (*p* < 0.01 for both)
Jang et al. [40], 2021 (South Korea)	64	-NCCN resectability criteria including extent of soft tissue contacting arteries and veins, depth of soft tissue contacting arteries and veins, contrast enhancement of the tumor, and of soft tissue surrounding arteries and veins, and tumor size.	-Chemo	2 GI radiologists	-Only low contrast enhancement of the soft tissue contacting the artery (£ 46.4 HU) was significantly associated with R0 resection (adjusted odds ratio = 7.4; *p* = 0.01).

Abbreviations: *Chemo*: chemotherapy alone; *CRT*: chemoradiation therapy; *DFS*: disease-free survival; *EBRT*: external beam radiation therapy; *GI*: gastrointestinal; HU, Hounsfield Unit; *NCCN*: National Comprehensive Cancer Network; *NPV:* negative predictive value; *OS*: overall survival; *PDAC:* pancreatic ductal adenocarcinoma; *PPV:* positive predictive value; *RECIST:* Response Evaluation Criteria in Solid Tumors; *R0*: microscopically margin-negative resection; *ypT*: pathologic T stage after neoadjuvant therapy. ** Resectable tumors: no distant metastases, no paraaortic nodal metastasis (>1 cm in short axis), no evidence of invasion of the celiac axis, superior mesenteric artery, hepatic artery, or superior mesenteric vein–portal vein confluence. Arterial invasion: any direct tumor-to-vessel contiguity, even if it was less than 50%. Venous invasion: tumor-to-vessel circumferential > 50% >2–3 cm in length.

**Table 3:** Summary of studies to date that have assessed the use of radiomics in conjunction with CT to evaluate PDAC treatment response assessment following neoadjuvant therapy.

Author, Year (Country)	No. of Patients	Type of Neoadjuvant Therapy	Semantic Imaging Features or Laboratory Features Used for Comparison or Combination with Radiomic Features	Segmentation	Feature Extraction Software	Main Results
Chen et al. [29], 2017 (U.S.)	20	-CRT	None	Manual, ROIs containing the pancreas head	In-house MATLAB	-Changes in mean histograms of CT number (MCTN), standard deviation (SD), skewness, and kurtosis were associated with good vs. poor pathologic response (*p* = 0.046, 0.058, 0.042, and 0.12, respectively).
Ciaravino et al. [46], 2018 (Italy)	31	-Chemo -CRT	None	Manual, ROIs containing the tumor from primary staging and restaging CT	MaZda	-Of the texture features that were investigated, only kurtosis was significantly different between primary staging and restaging CT (*p* = 0.0046) and was indicative of tumor downstaging.
Kim et al. [37], 2019 (South Korea)	45	-Chemo -CRT	Resectability status based on NCCN criteria, CA 19-9	Manual, ROIs containing the tumor from primary staging and restaging CT	MISSTA	-CA 19-9 nor any of the texture features at primary staging were significantly associated with R0 resection. -However, several subtracted texture values (i.e., between primary staging and restaging) were significantly associated with R0 resection, including lower subtracted value of surface area (HR 1.077, *p* = 0.011), higher subtracted values of GLCM IDM (HR 0.000, *p* = 0.005) and GLCM contrast (HR 0.982, *p* = 0.012). -Also, the higher subtracted value of entropy (HR 0.159, *p* = 0.005) and lower subtracted value of GLCM entropy (HR 10.235, *p* = 0.036) were associated with improved overall survival.
Nasief et al. [47], 2020 (U.S.)	24 (672 CT)	-CRT	CA 19-9	Manual, ROIs containing the tumor	IBEX	-The C-index for the prediction of pathologic response was 0.69 for CA 19.9 alone, which improved to 0.87 for the combination of CA 19-9 + delta radiomic features. -Decrease in CA19-9 levels and delta radiomic features were also significantly associated with survival (*p* = 0.031 and 0.001, respectively).
Borhani et al. [48], 2020 (U.S.)	39	-Chemo -CRT	None	Manual, ROIs containing the tumor from primary staging and restaging CT	TedRAD	-Higher mean perfusion parameter values at primary staging had higher odds of a favorable pathologic response (OR = 1.06; 95% CI, 1.002–1.12). -The Cox model containing three texture features was significantly associated with disease-free survival (*p* = 0.001).
Rigiroli et al. [49], 2021 (U.S.)	194	-Chemo -CRT	Resectability status based on NCCN criteria	Semi-automatic, 3D VOIs containing the tumor and perivascular tissue surrounding the SMA	Python	-The model containing five perivessel and tumor radiomic features had an AUC of 0.71 to determine tumor involvement of the SMA, whereas resectability status based on NCCN criteria had an AUC of 0.54.

Abbreviations: *AUC*: area under the curve; *CA*: carbohydrate antigen; *CRT*: chemoradiotherapy; *GLCM:* Grey level co-occurrence matrices; *HR*: Hazard ratio; *IBEX:* Imaging Biomarker Explorer; *IDM*: inverse difference moment; *MISSTA*: Medical Imaging Software and Texture Analysis; *NCCN*: National Comprehensive Cancer Network; *OR:* odds ratio; *ROI*: region of interest; *SD*: Standard Deviation; *SMA:* superior mesenteric artery; *VOI*; volume of interest.

## References

[1] Rahib L., Wehner M.R., Matrisian L.M., Nead K.T. (2021). Estimated Projection of US Cancer Incidence and Death to 2040. JAMA Netw. Open.

[2] National Cancer Institute (2023). Cancer Stat Facts: Pancreatic Cancer.

[3] Barnes C.A., Chavez M.I., Tsai S., Aldakkak M., George B., Ritch P.S., Dua K., Clarke C.N., Tolat P., Hagen C. (2019). Survival of patients with borderline resectable pancreatic cancer who received neoadjuvant therapy and surgery. Surgery.

[4] Ferlay J., Ervik M., Lam F., Colombet M., Mery L., Piñeros M., Znaor A., Soerjomataram I., Bray F. Global Cancer Observatory: Cancer Today; International Agency for Research on Cancer: Lyon, France. https://gco.iarc.fr/today.

[5] Ferlay J., Partensky C., Bray F. (2016). More deaths from pancreatic cancer than breast cancer in the EU by 2017. Acta Oncol..

[6] Rose J.B., Rocha F.G., Alseidi A., Biehl T., Moonka R., Ryan J.A., Lin B., Picozzi V., Helton S. (2014). Extended neoadjuvant chemotherapy for borderline resectable pancreatic cancer demonstrates promising postoperative outcomes and survival. Ann. Surg. Oncol..

[7] McGuire S. (2016). World Cancer Report 2014. Geneva, Switzerland: World Health Organization, International Agency for Research on Cancer, WHO Press, 2015. Adv. Nutr..

[8] Di Sebastiano P., Grottola T., di Mola F.F. (2016). Borderline resectable pancreatic cancer and the role of neoadjuvant chemoradiotherapy. Updates Surg..

[9] Soriano A., Castells A., Ayuso C., Ayuso J.R., de Caralt M.T., Ginès M.A., Real M.I., Gilabert R., Quintó L., Trilla A. (2004). Preoperative staging and tumor resectability assessment of pancreatic cancer: Prospective study comparing endoscopic ultrasonography, helical computed tomography, magnetic resonance imaging, and angiography. Am. J. Gastroenterol..

[10] Janssen Q.P., Buettner S., Suker M., Beumer B.R., Addeo P., Bachellier P., Bahary N., Bekaii-Saab T., Bali M.A., Besselink M.G. (2019). Neoadjuvant FOLFIRINOX in Patients with Borderline Resectable Pancreatic Cancer: A Systematic Review and Patient-Level Meta-Analysis. J. Natl. Cancer Inst..

[11] Kulkarni N.M., Mannelli L., Zins M., Bhosale P.R., Arif-Tiwari H., Brook O.R., Hecht E.M., Kastrinos F., Wang Z.J., Soloff E.V. (2020). White paper on pancreatic ductal adenocarcinoma from society of abdominal radiology’s disease-focused panel for pancreatic ductal adenocarcinoma: Part II, update on imaging techniques and screening of pancreatic cancer in high-risk individuals. Abdom. Radiol..

[12] Takahashi S., Ohno I., Ikeda M., Konishi M., Kobayashi T., Akimoto T., Kojima M., Morinaga S., Toyama H., Shimizu Y. (2022). Neoadjuvant S-1 With Concurrent Radiotherapy Followed by Surgery for Borderline Resectable Pancreatic Cancer: A Phase II Open-label Multicenter Prospective Trial (JASPAC05). Ann. Surg..

[13] Tamm E.P., Loyer E.M., Faria S., Raut C.P., Evans D.B., Wolff R.A., Crane C.H., Dubrow R.A., Charnsangavej C. (2006). Staging of pancreatic cancer with multidetector CT in the setting of preoperative chemoradiation therapy. Abdom. Imaging.

[14] National Comprehensive Cancer Network (2023). NCCN Clinical Practice Guidelines in Oncology (NCCN Guidelines^®^). Pancreatic Adenocarcinoma. Version 2..

[15] Al-Hawary M.M., Francis I.R., Chari S.T., Fishman E.K., Hough D.M., Lu D.S., Macari M., Megibow A.J., Miller F.H., Mortele K.J. (2014). Pancreatic ductal adenocarcinoma radiology reporting template: Consensus statement of the Society of Abdominal Radiology and the American Pancreatic Association. Radiology.

[16] Windsor J.A., Barreto S.G. (2017). The concept of ‘borderline resectable’ pancreatic cancer: Limited foundations and limited future?. J. Gastrointest. Oncol..

[17] Balthazar E.J. (2005). Pancreatitis associated with pancreatic carcinoma. Preoperative diagnosis: Role of CT imaging in detection and evaluation. Pancreatology.

[18] Neoptolemos J.P., Stocken D.D., Bassi C., Ghaneh P., Cunningham D., Goldstein D., Padbury R., Moore M.J., Gallinger S., Mariette C. (2010). Adjuvant chemotherapy with fluorouracil plus folinic acid vs gemcitabine following pancreatic cancer resection: A randomized controlled trial. JAMA.

[19] Conroy T., Castan F., Lopez A., Turpin A., Ben Abdelghani M., Wei A.C., Mitry E., Biagi J.J., Evesque L., Artru P. (2022). Five-Year Outcomes of FOLFIRINOX vs Gemcitabine as Adjuvant Therapy for Pancreatic Cancer: A Randomized Clinical Trial. JAMA Oncol..

[20] Versteijne E., van Dam J.L., Suker M., Janssen Q.P., Groothuis K., Akkermans-Vogelaar J.M., Besselink M.G., Bonsing B.A., Buijsen J., Busch O.R. (2022). Neoadjuvant Chemoradiotherapy Versus Upfront Surgery for Resectable and Borderline Resectable Pancreatic Cancer: Long-Term Results of the Dutch Randomized PREOPANC Trial. J. Clin. Oncol..

[21] Dhir M., Malhotra G.K., Sohal D.P.S., Hein N.A., Smith L.M., O’Reilly E.M., Bahary N., Are C. (2017). Neoadjuvant treatment of pancreatic adenocarcinoma: A systematic review and meta-analysis of 5520 patients. World J. Surg. Oncol..

[22] Brown Z.J., Heh V., Labiner H.E., Brock G.N., Ejaz A., Dillhoff M., Tsung A., Pawlik T.M., Cloyd J.M. (2022). Surgical resection rates after neoadjuvant therapy for localized pancreatic ductal adenocarcinoma: Meta-analysis. Br. J. Surg..

[23] Yang B., Chen K., Liu W., Long D., Wang Y., Liu X., Ma Y., Tian X., Yang Y. (2023). The benefits of neoadjuvant therapy for patients with resectable pancreatic cancer: An updated systematic review and meta-analysis. Clin. Exp. Med..

[24] Ratnayake B., Savastyuk A.Y., Nayar M., Wilson C.H., Windsor J.A., Roberts K., French J.J., Pandanaboyana S. (2020). Recurrence Patterns for Pancreatic Ductal Adenocarcinoma after Upfront Resection Versus Resection Following Neoadjuvant Therapy: A Comprehensive Meta-Analysis. J. Clin. Med..

[25] Lindemann J., du Toit L., Kotze U., Bernon M., Krige J., Jonas E. (2021). Survival equivalence in patients treated for borderline resectable and unresectable locally advanced pancreatic ductal adenocarcinoma: A systematic review and network meta-analysis. HPB.

[26] Versteijne E., de Hingh I., Homs M.Y.V., Intven M.P.W., Klaase J.M., van Santvoort H.C., de Vos-Geelen J., Wilmink J.W., van Tienhoven G. (2021). Neoadjuvant Treatment for Resectable and Borderline Resectable Pancreatic Cancer: Chemotherapy or Chemoradiotherapy?. Front. Oncol..

[27] Motoi F., Unno M. (2020). Adjuvant and neoadjuvant treatment for pancreatic adenocarcinoma. Jpn. J. Clin. Oncol..

[28] Ferrone C.R., Marchegiani G., Hong T.S., Ryan D.P., Deshpande V., McDonnell E.I., Sabbatino F., Santos D.D., Allen J.N., Blaszkowsky L.S. (2015). Radiological and surgical implications of neoadjuvant treatment with FOLFIRINOX for locally advanced and borderline resectable pancreatic cancer. Ann. Surg..

[29] Chen X., Oshima K., Schott D., Wu H., Hall W., Song Y., Tao Y., Li D., Zheng C., Knechtges P. (2017). Assessment of treatment response during chemoradiation therapy for pancreatic cancer based on quantitative radiomic analysis of daily CTs: An exploratory study. PLoS ONE.

[30] Barreto S.G., Loveday B., Windsor J.A., Pandanaboyana S. (2019). Detecting tumour response and predicting resectability after neoadjuvant therapy for borderline resectable and locally advanced pancreatic cancer. ANZ J. Surg..

[31] Katz M.H., Fleming J.B., Bhosale P., Varadhachary G., Lee J.E., Wolff R., Wang H., Abbruzzese J., Pisters P.W., Vauthey J.N. (2012). Response of borderline resectable pancreatic cancer to neoadjuvant therapy is not reflected by radiographic indicators. Cancer.

[32] Kim Y.E., Park M.S., Hong H.S., Kang C.M., Choi J.Y., Lim J.S., Lee W.J., Kim M.J., Kim K.W. (2009). Effects of neoadjuvant combined chemotherapy and radiation therapy on the CT evaluation of resectability and staging in patients with pancreatic head cancer. Radiology.

[33] Cassinotto C., Cortade J., Belleannée G., Lapuyade B., Terrebonne E., Vendrely V., Laurent C., Sa-Cunha A. (2013). An evaluation of the accuracy of CT when determining resectability of pancreatic head adenocarcinoma after neoadjuvant treatment. Eur. J. Radiol..

[34] Wagner M., Antunes C., Pietrasz D., Cassinotto C., Zappa M., Sa Cunha A., Lucidarme O., Bachet J.B. (2017). CT evaluation after neoadjuvant FOLFIRINOX chemotherapy for borderline and locally advanced pancreatic adenocarcinoma. Eur. Radiol..

[35] Amer A.M., Zaid M., Chaudhury B., Elganainy D., Lee Y., Wilke C.T., Cloyd J., Wang H., Maitra A., Wolff R.A. (2018). Imaging-based biomarkers: Changes in the tumor interface of pancreatic ductal adenocarcinoma on computed tomography scans indicate response to cytotoxic therapy. Cancer.

[36] Marchegiani G., Todaro V., Boninsegna E., Negrelli R., Sureka B., Bonamini D., Salvia R., Manfredi R., Pozzi Mucelli R., Bassi C. (2018). Surgery after FOLFIRINOX treatment for locally advanced and borderline resectable pancreatic cancer: Increase in tumour attenuation on CT correlates with R0 resection. Eur. Radiol..

[37] Kim B.R., Kim J.H., Ahn S.J., Joo I., Choi S.Y., Park S.J., Han J.K. (2019). CT prediction of resectability and prognosis in patients with pancreatic ductal adenocarcinoma after neoadjuvant treatment using image findings and texture analysis. Eur. Radiol..

[38] Wei D., Zaid M.M., Katz M.H., Prakash L.R., Kim M., Tzeng C.D., Lee J.E., Agrawal A., Rashid A., Wang H. (2021). Clinicopathological correlation of radiologic measurement of post-therapy tumor size and tumor volume for pancreatic ductal adenocarcinoma. Pancreatology.

[39] Perri G., Prakash L., Wang H., Bhosale P., Varadhachary G.R., Wolff R., Fogelman D., Overman M., Pant S., Javle M. (2021). Radiographic and Serologic Predictors of Pathologic Major Response to Preoperative Therapy for Pancreatic Cancer. Ann. Surg..

[40] Jang J.K., Byun J.H., Kang J.H., Son J.H., Kim J.H., Lee S.S., Kim H.J., Yoo C., Kim K.P., Hong S.M. (2021). CT-determined resectability of borderline resectable and unresectable pancreatic adenocarcinoma following FOLFIRINOX therapy. Eur. Radiol..

[41] Aerts H.J., Velazquez E.R., Leijenaar R.T., Parmar C., Grossmann P., Carvalho S., Bussink J., Monshouwer R., Haibe-Kains B., Rietveld D. (2014). Decoding tumour phenotype by noninvasive imaging using a quantitative radiomics approach. Nat. Commun..

[42] Attiyeh M.A., Chakraborty J., Doussot A., Langdon-Embry L., Mainarich S., Gönen M., Balachandran V.P., D’Angelica M.I., DeMatteo R.P., Jarnagin W.R. (2018). Survival Prediction in Pancreatic Ductal Adenocarcinoma by Quantitative Computed Tomography Image Analysis. Ann. Surg. Oncol..

[43] Khalvati F., Zhang Y., Baig S., Lobo-Mueller E.M., Karanicolas P., Gallinger S., Haider M.A. (2019). Prognostic Value of CT Radiomic Features in Resectable Pancreatic Ductal Adenocarcinoma. Sci. Rep..

[44] Shur J.D., Doran S.J., Kumar S., Ap Dafydd D., Downey K., O’Connor J.P.B., Papanikolaou N., Messiou C., Koh D.M., Orton M.R. (2021). Radiomics in Oncology: A Practical Guide. Radiographics.

[45] Horvat N., Miranda J., El Homsi M., Peoples J.J., Long N.M., Simpson A.L., Do R.K.G. (2021). A primer on texture analysis in abdominal radiology. Abdom. Radiol..

[46] Ciaravino V., Cardobi N., De Robertis R., Capelli P., Melisi D., Simionato F., Marchegiani G., Salvia R., D’Onofrio M. (2018). CT Texture Analysis of Ductal Adenocarcinoma Downstaged After Chemotherapy. Anticancer Res..

[47] Nasief H., Hall W., Zheng C., Tsai S., Wang L., Erickson B., Li X.A. (2019). Improving Treatment Response Prediction for Chemoradiation Therapy of Pancreatic Cancer Using a Combination of Delta-Radiomics and the Clinical Biomarker CA19-9. Front. Oncol..

[48] Borhani A.A., Dewan R., Furlan A., Seiser N., Zureikat A.H., Singhi A.D., Boone B., Bahary N., Hogg M.E., Lotze M. (2020). Assessment of Response to Neoadjuvant Therapy Using CT Texture Analysis in Patients with Resectable and Borderline Resectable Pancreatic Ductal Adenocarcinoma. AJR Am. J. Roentgenol..

[49] Rigiroli F., Hoye J., Lerebours R., Lafata K.J., Li C., Meyer M., Lyu P., Ding Y., Schwartz F.R., Mettu N.B. (2021). CT Radiomic Features of Superior Mesenteric Artery Involvement in Pancreatic Ductal Adenocarcinoma: A Pilot Study. Radiology.

[50] Soloff E.V., Al-Hawary M.M., Desser T.S., Fishman E.K., Minter R.M., Zins M. (2022). Imaging Assessment of Pancreatic Cancer Resectability After Neoadjuvant Therapy: *AJR* Expert Panel Narrative Review. AJR Am. J. Roentgenol..

